# The Use of Calcium and Magnesium to Prevent Neurotoxicity in Patients Receiving Oxaliplatin

**Published:** 2015-05-01

**Authors:** Karon Martyn, Emily Petito

**Affiliations:** Fox Chase Cancer Center, Philadelphia, Pennsylvania, and Sidney Kimmel Comprehensive Cancer Center, Johns Hopkins Medicine, Baltimore, Marylan

A groundbreaking oral abstract was presented by Charles Loprinzi at the 2013 annual meeting of the American Society of Clinical Oncology (ASCO), addressing the controversial issue of using magnesium and calcium to prevent oxaliplatin-induced sensory neuropathy. Results of the study were subsequently published in the *Journal of Clinical Oncology* [Loprinzi et al., 2014]). This practice has varied among many institutions for nearly a decade due to the lack of a clear message in the literature to support or contest this practice.

The phase III randomized, placebo-controlled, double-blinded study was conducted by Loprinzi et al. with the primary objective of determining whether the use of 1 g of calcium gluconate and 1 g of magnesium sulfate reduced cumulative sensory neuropathy. The trial included 353 total patients being treated with adjuvant 5-fluorouracil, oxaliplatin, and leucovorin (FOLFOX) for colon cancer. Patients were divided among three arms. The first arm included 118 patients who received calcium and magnesium before and after oxaliplatin. The second arm included 116 patients who received calcium and magnesium before oxaliplatin, followed by placebo. The third arm included 119 patients who received placebo before and after oxaliplatin.

The primary endpoint used the European Organisation for Research and Treatment of Cancer (EORTC) QLQ-CIPN20 scale to measure sensory neuropathy. The Common Terminology Criteria for Adverse Events (CTCAE) 4.0 and an oxaliplatin-specific neurotoxicity scale were used to measure secondary endpoints of median days to grade 2 sensory neurotoxicity. Acute sensory neuropathy was also assessed for 5 days after oxaliplatin.

The study concluded that the use of Ca/Mg to prevent sensory neuropathy did not provide benefit to patients from an acute or cumulative neuropathy standpoint. There was no difference found among all arms when using the oxaliplatin-specific neuropathy scale, the CTCAE scale, or the QLQ-CIPN20 scale with respect to median days to development of grade 2 sensory neuropathy (Loprinzi et al., 2014).

## RETROSPECTIVE STUDIES

In a review of the literature leading up to this groundbreaking study, multiple retrospective and prospective studies have been conducted over the years (see Table). Upon review of the retrospective data, a study conducted by Gamelin et al. published in 2004 was one of the first pivotal studies that addressed this conundrum. This trial included a total of 161 patients treated with three different chemotherapy regimens using oxaliplatin. The doses varied from 85 mg/m² every 2 weeks, 100 mg/m² every 2 weeks, and 130 mg/m² every 3 weeks. Patients received treatment from 1996 to 2000. Sixty-five patients were included in the control arm. Ninety-six patients received 1 g of calcium gluconate and 1 g of magnesium sulfate, before and after oxaliplatin.

**Table 1:**
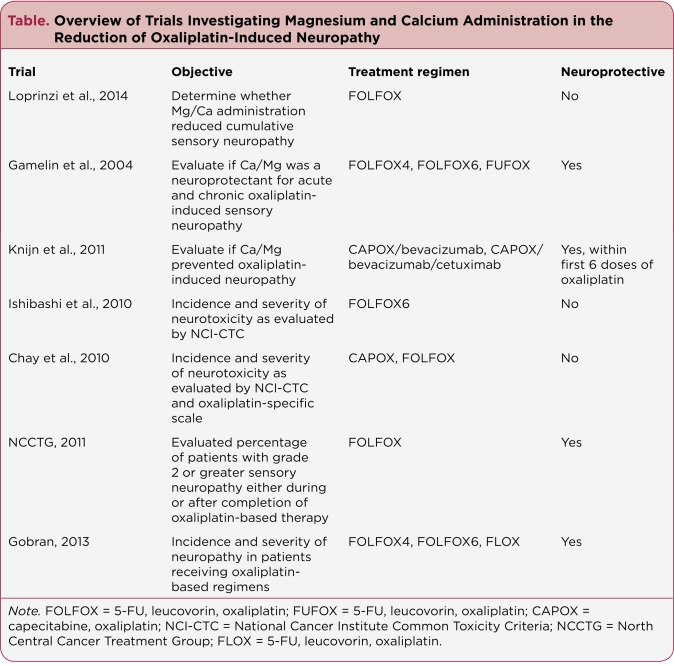
Overview of Trials Investigating Magnesium and Calcium Administration in the Reduction of Oxaliplatin-Induced Neuropathy

The main objective of the study was to evaluate whether calcium and magnesium proved to be a neuroprotectant for both acute and chronic oxaliplatin-induced sensory neuropathy. Neuropathy was assessed using the National Cancer Institute Common Toxicity Criteria version 1 (NCI-CTC) and an oxaliplatin-related neurotoxicity scale. Scales were used to measure weekly and biweekly sensory neuropathy according to the frequency of oxaliplatin administration. Treatment delays and dose modifications were made according to the standard of practice. Twenty-nine patients received FOLFOX4 (oxaliplatin dose of 85/mg/m² every 2 weeks), 25 patients received FOLFOX6 (oxaliplatin dose of 100 mg/m² every 2 weeks), and 42 patients received FUFOX (oxaliplatin dose of 130 mg/m² every 3 weeks) with calcium and magnesium pre- and posttreatment. The study supported the use of calcium and magnesium pre- and post-oxaliplatin in an effort to reduce incidence and severity of acute symptoms, especially at the 85 mg/m² dose. According to Gamelin et al. (2004), only 4% of patients withdrew due to neurotoxicity in the Ca/Mg group vs. 31% in the control group (*p* = .000003). Also, 20% of patients in the Ca/Mg group had neuropathy vs. 45% in the control group (p = .0003). Limitations included the fact that this was a retrospective study that was neither randomized nor blinded.

Another retrospective study by Knijnet al. published in 2011 examined data obtained in the phase III CAIRO2 study. CAIRO2 analyzed advanced colorectal patients who were not previously treated. The primary goal of the Knijnet al. group was to analyze if there was any utility with the prophylactic use of Ca/Mg to prevent oxaliplatin-induced neuropathy.

A total of 755 individuals were randomized to receive CAPOX/bevacizumab (CB) or CAPOX/bevacizumab/cetuximab (CBC); 732 of these patients were included in the review. In the experimental group, 551 patients received calcium and magnesium over 15 minutes before and after oxaliplatin. Of those individuals, 270 were in the CB arm, and 281 were in the CBC arm. The control group consisted of a total of 181 patients, with 96 patients in the CB group and 85 patients in the CBC group. Individuals with baseline neuropathy above grade 1 were not included. To exclude patients who might have received Ca/Mg for secondary measures, those included in the Knijnet al. study had to have received Ca/Mg at least with the first dose of oxaliplatin. Patients received a maximum of six doses of oxaliplatin.

Neurotoxicity was measured by the NCI-CTC version 3 scale. Subgroups were formed to examine early vs. late neurotoxicity. Early neurotoxicity was described as neuropathy that occurred within the first six doses of oxaliplatin. Late neurotoxicity was that seen in patients who experienced neuropathy at the end of six doses of oxaliplatin. Neuropathy was measured by all grades and further broken down to grades 1, 2, and 3.

A notable reduction in sensory neuropathy was found in the all-grade group (*p* = .02), who received Ca/Mg before and after oxaliplatin (Knijnet al., 2011). There was also a notable finding in the early neurotoxicity group who received Ca/Mg (*p* = .002). There was no difference found in individuals experiencing a grade 2 or higher neurotoxicity.

One limitation of this study is that it was retrospective. Therefore, there is a threat that the data obtained may have been biased. However, the policy of utilizing Ca/Mg infusions was consistent among all facilities included in the study. On the positive side, this study consisted of a large sample size. However, the control arm was significantly smaller than the Ca/Mg arm. The study was also limited by treatments to a maximum of 6 oxaliplatin infusions, in accordance with the OPTIMOX study. Therefore, the findings do not support the use of Ca/Mg beyond six oxaliplatin infusions.

In the end, this study did not find that Ca/Mg provided neuroprotection in patients who suffered from grade 2 or higher neuropathy. However, it did support the use of Ca/Mg to reduce early neuropathy or neuropathy occurring in the first six cycles of oxaliplatin. Furthermore, Knijnet al. did not find that the use of calcium and magnesium led to an inferior overall survival or response rate when used with oxaliplatin.

## PROSPECTIVE RANDOMIZED CONTROLLED TRIALS

There have been four published prospective randomized controlled trials to date examining the impact of calcium and magnesium infusions on neurotoxicity with oxaliplatin administration. The first study was conducted by Ishibashi et al. (2010) and included patients who had either unresectable metastatic colorectal cancer or who had already undergone resection for metastatic lesions. All patients were treated with oxaliplatin 85 mg/m² as indicated by the modified FOLFOX6 protocol. For those patients with unresectable disease, treatment continued until disease progression or unacceptable toxicities. In the group with resected disease, treatment was continued for six cycles.

The primary endpoint of the study was the incidence and severity of neurotoxicity as evaluated by the NCI-CTC and the Debiopharm Neurotoxicity Scale criteria. Neurotoxicity assessment was completed by both nurses and pharmacists at the chemotherapy center. Secondary endpoints of the study were antitumor activity, progression-free survival, and plasma levels of platinum. The study enrolled 33 patients starting mFOLFOX6 between October 2006 and September 2007.

There was no statistically significant difference in the efficacy of treatment between the two groups as well as no significant difference in plasma platinum concentrations between the groups. Lastly, they found no difference in the incidence of grade 1, 2, or 3 neurotoxicity after the completion of the sixth cycle of treatment. The study initially intended to accrue 35 patients per group. Study enrollment, however, was prematurely discontinued based on the interim analysis by an unsubstantiated data monitoring committee of the CONcePT study (Grothey, 2008), which reported that patients receiving calcium and magnesium infusions had poorer treatment outcomes compared to those receiving placebo.

These results were later determined to be erroneous by an independent, blinded review of the radiologic scans from the CONcePT study, which determined that the response rate was in fact lower in the group receiving placebo vs. the group receiving calcium and magnesium infusions. While the study by Ishibashi et al. (2010) indicated that calcium and magnesium infusions have no impact on the incidence of neurotoxicity, it was limited by small sample size and premature closing.

A single-institution, blinded, randomized phase II study was conducted by Chay et al. (2010) from October 2005 through June 2007. This placebo-controlled study included patients with colorectal cancer receiving treatment for metastatic disease as well as those receiving adjuvant therapy following curative resection. Patients were evaluated for neurotoxicity using the NCI-CTC and an oxaliplatin-specific scale following every cycle of chemotherapy. Patients were also evaluated by nerve conduction studies at baseline, and after 4, 8, and 12 cycles of chemotherapy. A total of 27 patients were enrolled and patients received either CAPOX with oral capecitabine 1,000 mg/m² twice daily days 1–14 of a 21-day cycle and oxaliplatin 130 mg/m² on day 1 of a 21-day cycle or FOLFOX4 with doses of oxaliplatin beginning at 85 mg/m². The majority of patients on both treatment and placebo arms received CAPOX, with only 5 of the 27 patients receiving FOLFOX. There was no statistically significant difference in the incidence of either acute or cumulative sensory neuropathy between the two arms (Chay et al., 2010). This study was also prematurely discontinued based on the preliminary results of the CONcePT study described previously (Grothey, 2008).

The North Central Cancer Treatment Group (NCCTG) published results from the multicenter trial N04C7 in 2011. This study included only patients receiving adjuvant FOLFOX and excluded patients with metastatic disease (Grothey et al., 2011). The primary endpoint was the percentage of patients with grade 2 or greater sensory neuropathy either during or after completion of oxaliplatin-based therapy. Neurotoxicity was evaluated by the NCI-CTC criteria as well as the oxaliplatin-specific sensory neurotoxicity scale and patient questionnaire. A total of 102 patients were enrolled in this study. Similar to the previous two studies discussed, this study was discontinued prematurely based on the erroneous results of the CONcePT trial. Unlike the previous two studies, this trial did indicate a statistically significant decrease in the incidence of chronic, cumulative grade 2 or greater neuropathy. Overall 22% of patients had grade 2 sensory neurotoxicity by the NCI-CTC scale in the calcium and magnesium group as compared to 38.5% in the placebo arm. Interestingly, there was no difference in the effect of calcium and magnesium on acute, cold-induced neurotoxicity (Grothey, 2011).

A study published in 2013 in the *Chinese-German Journal of Clinical Oncology* has also supported findings of a statistically significant reduction in neurotoxicity in patients receiving calcium and magnesium with oxaliplatin infusions (Gobran, 2013). The study was conducted from July 2008 through February 2011 and enrolled 30 patients to the calcium/magnesium treatment arm and 30 patients to the placebo arm. All patients were receiving an oxaliplatin-containing regimen as adjuvant treatment and received either FOLFOX4, mFOLFOX6, or FLOX. Neuropathy was assessed using the NCI-CTCAE version 3.0 criteria. Results concluded that 23.3% of patients receiving calcium and magnesium developed neuropathy by the completion of therapy, vs. 46.6% in the placebo arm, a statistically significant difference (Gobran, 2013).

## CONCLUSION

Oxaliplatin-induced neuropathy continues to be difficult to treat and prevent and poses a significant threat to a colorectal cancer patient’s quality of life. The question of whether calcium and magnesium infusions can reduce the incidence and severity of this toxicity has been addressed in numerous studies throughout the past decade, with conflicting results. Until Loprinzi’s group presented their findings at the ASCO annual meeting in 2013, the studies were often limited by their small sample size, preliminary closing, or retrospective nature. Due to the rigorous manner of the 2013 abstract by Loprinzi et al., which found no statistically significant difference in oxaliplatin-induced neurotoxicity, we would not recommend that patients receive calcium and magnesium infusions with oxaliplatin treatments.
